# “It’s a Little Bit Tricky”: Results from the POLYamorous Childbearing and Birth Experiences Study (POLYBABES)

**DOI:** 10.1007/s10508-021-02025-5

**Published:** 2021-06-01

**Authors:** Samantha Landry, Erika Arseneau, Elizabeth K. Darling

**Affiliations:** 1 Midwifery Education Program, McMaster University, HSC-4H24K, 1280 Main St. W., Hamilton, ON L8S 4K1 Canada; 2 Department of Obstetrics & Gynecology, McMaster University, Hamilton, ON Canada

**Keywords:** Polyamory, Pregnancy, Birth, Non-monogamy, POLYBABES

## Abstract

The number of polyamorous people in Canada is growing steadily, and many polyamorous people are of childbearing age and report living with children. Experiences of polyamorous families, particularly those related to pregnancy and childbirth, have thus far been underrepresented in the literature. The POLYamorous Childbearing and Birth Experiences Study (POLYBABES) sought to explore the pregnancy and birth experiences of polyamorous people. Having previously reported findings relating to experiences with the health system and healthcare providers, this article specifically focuses on the social aspects of polyamorous families’ experiences. We explored the impact of polyamory on one’s self identity, relationship structures, and experiences navigating the social world. Anyone who self-identified as polyamorous during pregnancy and birth, gave birth in Canada within 5 years, and received some prenatal care was eligible to participate in this study. Participants were recruited through social media and interviewed online or in person. Twenty-four participants were interviewed (11 birthing people and 13 of their partners). Thematic analysis was used to explore the data, and four primary themes were identified: deliberately planning families, more is more, presenting polyamory, and living in a mononormative world. Each theme was further broken down into a number of sub-themes. We also collaborated with research participants to create a glossary of terms. By exploring the pregnancy and birth experiences of polyamorous families and focusing on participant voices, this research adds to the limited research on polyamorous families and contributes to the process of breaking down stigma associated with alternative family structures. Further, by creating an accessible glossary of terms, researchers and lay persons alike have been given access to a meaningful resource.

## Introduction

Polyamorous individuals, a subgroup of the larger population of people who identify as consensually non-monogamous, represent a growing group of North Americans. Recent surveys estimate approximately one in five single Americans having participated in a consensually non-monogamous relationship at some point in their lives (Haupert et al., 2017). Similar estimates have come from Canadian-based studies (Fairbrother et al., 2019). Not all people who engage in consensual non-monogamy are polyamorous; however, specific prevalence estimates of polyamory are limited and often subject to methodological limitations (Haupert et al., 2017). Research has shown that many members of the polyamory community live in family arrangements with one or more partners and may parent children. For instance, a study conducted by the Canadian Research Institute for Law and the Family in 2016 surveyed Canadians about polyamory and found that 68% (*n* = 372) of respondents were involved in a polyamorous relationship at the time of the survey and that 23.2% (*n* = 127) resided full-time with at least one child 18 years old or younger. Further, the majority of survey respondents (75%) were between the ages of 25 and 44, which are common childbearing years (Boyd, 2017).

Research on polyamory has been historically lacking from scientific literature. However, since 2010 the concept has become more mainstream in research literature (Klesse, 2019) and in society. As social awareness of polyamory has grown, stigma against non-monogamous relationships has become more transparent. For instance, recent thematic analysis of lay perceptions of polyamory found reactions from the general population ranged from conceptualizing polyamory as valid and beneficial or acceptable to more negative views of perversion, deviance, and unsustainability (Séguin, 2019). With respect to polyamory and childrearing, people have often expressed opinions that polyamory is dangerous to children (Séguin, 2019). Researchers have noted that in situations where polyamorous parents are raising children, stigma is often more pronounced than if children were not involved (Pallotta-Chiarolli et al., 2020; Sheff, 2013).

While negative lay perceptions of polyamorous child rearing have often been documented (Séguin, 2019; Sheff, 2013), research has also suggested that there are some benefits to raising children in polyamorous family configurations (Goldfeder & Sheff, 2013; Klesse, 2019; Pain, 2020; Sheff, 2013, 2015). That being said, the lived experiences of family planning, pregnancy, and childbirth for polyamorous folks have largely gone unreported (Arseneau et al., 2019) and the general experiences of polyamorous families have thus far been underrepresented in the literature. Many have called for the volume of polyamorous research to increase and have recognized qualitative research as an optimal forum for doing so (Reczek, 2020).

### Research Aims

The main objective of the POLYamorous childBearing and Birth Experiences Study (POLYBABES) was to explore the experiences of polyamorous people in Canada during pregnancy and birth. Original objectives of the study included reporting on lived experiences, analyzing barriers to healthcare, and disseminating findings in a meaningful and accessible way. We sought to do this through semi-structured interviews where conversation was primarily guided by participants. Our interviews provided illumination of two different dimensions of the reported experiences: the medical and the social. We previously reported the experiences of polyamorous families with the healthcare system and healthcare providers (Arseneau et al., 2019). In this article, we share the social experience of our polyamorous participants, focusing on relationships, social perceptions and identity.

## Method

### Participants

Study inclusion criteria required participants to self-identify as polyamorous during the time of their pregnancy, to have given birth within the previous 5 years, and to have received some prenatal care in Canada (from a general practitioner [GP], obstetrician [OB], or registered midwife [RM]). We invited birthing folks and any willing partner(s) to be interviewed online or in person, either together or separately. We recruited participants primarily through social media (Facebook) in a series of postings targeting midwifery groups and polyamorous social groups across Canada. We contacted administrators of polyamorous social groups on Facebook throughout the country and asked them to disseminate our recruitment ad. Several midwifery associations across the country also posted the ad online and in clinics. Additionally, some members of the research team shared the recruitment poster through their own social media platforms. Participants were recruited through convenience and snowballing techniques. Participants were encouraged to reach out to any friends or acquaintances who might meet inclusion criteria for participation.

### Measures

#### Demographic Questionnaire

Prior to being interviewed and after providing informed consent, participants were asked to complete a short semi-structured online demographic questionnaire hosted through Qualtrics. This questionnaire consisted of open-ended questions regarding sexual orientation, gender, childbirth experience, and household contributors. Questions about ethnic background and education were modelled after Statistics Canada questions. Participants were also asked for consent to participate in future research projects and to be contacted for follow-up about this project.

#### Interview Guide

Two primary questions guided the interview process: “Can you tell us about your relationship structures now and during your pregnancy and birth?” and “Please tell us about your pregnancy and birth experience.” From these initial questions, further probing questions were asked regarding disclosure, experiences with healthcare providers, future pregnancy intentions, and term definitions. As part of the research study, at the onset of each interview participants were informed that the researchers sought to create a glossary of terms using participant definitions and experiences and were asked to define any polyamory related jargon they discussed. We also asked each participant to define the term “polyamory” both to allow us to appreciate similarities and differences in how polyamory was conceptualized by participants and to build a comprehensive definition for the intended glossary.

### Procedure and Thematic Analysis

Once the online demographic questionnaire and research ethics forms were completed, we scheduled interviews with birthing persons and any interested partner(s). Online interviews took place over web-conferencing software (Zoom) and were recorded and saved locally. Audio recordings of in-person interviews were made and stored securely. All interviews were professionally transcribed and de-identified in the process.

We used Braun and Clark’s (2008) six step thematic analysis to explore the data. In step one, we familiarized ourselves with the data. Each of the interviewers created jot notes following each interview. Attempts were made to conceptualize broad themes across interviews as they occurred. As the majority of interviews (9 of 11) were conducted by both primary investigators, debriefing occurred after each. In step two, initial codes were generated from interview transcripts. We then conducted open coding using a line by line process. Coding was inductive rather than theoretical. Transcripts were coded by two co-investigators (EA and SL) using NVivo 12 software. The first transcript was coded simultaneously by both coders. The coders then each coded the second transcript independently and compared for consistency. Finally, the remaining transcripts were divided, and each was coded by a single investigator. After initial coding was complete, the co-investigators compared and contrasted codebooks for consistency and in order to collapse synonymous codes. Next, a tree was created using all generated codes which were then grouped into general categories from which themes were developed. These themes were then reviewed and further defined.

A glossary of terms was also created from terms identified and defined by participants. The initial glossary, which included terms, definitions, and supporting direct quotes, was sent to all study participants to review and edit. Several study participants provided feedback on the glossary by modifying existing definitions, adding new terms, and elaborating on existing terms. The glossary was then finalized and posted online at http://www.polybabes.ca to be used by clinicians, polyamorous folks, and the general population.

## Results

### Demographic Questionnaire

**Fig. 1:**
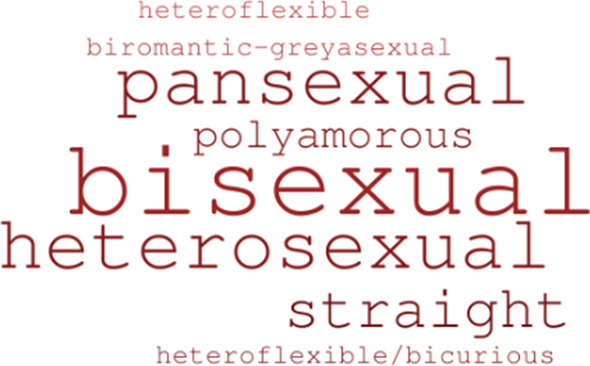
Sexual orientation

In total, 24 participants were interviewed: 11 birthing people and 13 of their partners. Participant ages ranged from 23 to 49 years with a mean age of 34 years. Most (81.8%) participants identified as White/European. Black/African/Caribbean and Aboriginal/First Nations backgrounds were endorsed by a small number of participants (9.1% each). Overall, study participants were highly educated with 81.8% having completed some level of university or college education, 13.6% having completed some college or university, and 4.5% having completed high school. Sexual orientation was posed as an open-ended question. Figure 1 captures responses with orientations most commonly reported in largest font and those least commonly reported in smallest font. All participants expressed interest in being contacted for follow-up to this study and for future studies.

Because of the small sample size and unique family structures of those interviewed, we have chosen not to provide more information about individual family compositions and age/race of individual participants in order to protect the anonymity of study participants. We have attempted to minimize identifiable demographic information within direct quotations when possible. Quotes have been organized in a system of letters (A–K) and numbers (1–4) where each letter denotes a family and each number an interviewed member of that family. All birthing persons have been coded as 1 (e.g., A1, B1, C1).

### Glossary of Terms

At the onset of each interview, participants were asked to contribute to a glossary of terms by defining any polyamory or consensual non-monogamy related jargon used in interviews (www.polybabes.ca/glossary). All participants were receptive to the creation of the glossary. In total, 30 terms were defined by one or more individuals. All study participants were asked to define polyamory in their own words and to expand on any variance. Definitions of polyamory ranged from concise to elaborate.

It is being involved in multiple loving romantic relationships with the joyful consent of everyone involved. (K1).

[Polyamory is] kind of a subset or a type of open relationship where the type of partners that people have and the types of multi-intimate (…) encounters or relationships that people have are not limited to being just sexual, that people want to and are allowed to have multiple loving, perhaps long-term relationships and (…) the idea is that it’s honest, above-board and it has the consent of people. (E2).

Though recruitment for the study encouraged self-defining as polyamorous (no prescribed definition was provided), collecting individual definitions from participants provided evidence that participant definitions were generally aligned. Most participant definitions included a focus on the openness that comes with polyamory. Participants described polyamory as “freedom,” “non-controlling,” “non-restrictive,” “ability to explore,” and largely focused on “potential.” The concept of capacity-for or theoretical-possibility-of relationships was mentioned frequently; polyamory, thus, is not based on the number of partners at a given time, but it is instead focused on the potential of having multiple partners. Additionally, several participants discussed the importance of being able to celebrate their partners’ happiness with other people. The focus was not only on their own capacity for multiple relationships, but also on their partners’ experiences of other partners.

### Family Structures

While analyzing and reporting on family structures was not an initial goal of the research, being aware of the varying dynamics of the families surveyed is important for conceptualizing their experiences. Research participants were asked to describe their family structures at the time of their pregnancy and at the time of the interview, which for some was several years later. Participants identified the contributors to their households and additional partners who may not have been involved with pregnancy and childrearing. Within most of the families interviewed, legal marital unions were present and were often (but not always) the biological contributors to the pregnancies. In other families, legal unions did not directly link with level of commitment or length of relationships. Several surveyed participants had one or more partner who chose not to participate in the research study; often, these other partners did not play active roles in childbearing or parenting. Though several participants explained that being actively involved in one or more relationships was not a prerequisite to being polyamorous, every participant interviewed had at least one partner both at the time of their pregnancy and at the time of the interview. Several participants expressed that their non-cohabitating partners played active parts in their child(ren)’s lives, while no participants identified cohabitating partners who were not parenting. Multiple interviewed families included children from previous relationships who were co-parented by the adults in their households. In two families, individuals interviewed shared custody of children who were not always present in the home.

As elucidated, relationship structures of the families surveyed differed widely. Households ranged from three to nine people and were occasionally dynamic. Not only did the number participants’ partners vary, but their relationship agreements did as well. Expressions of relationship structure varied among each family. Some participants expressed more of a hierarchical relationship structure (whether defined by legal unions, length of relationship or a variety of other factors), whereas others specifically strived for egalitarian relationships between all partners.

### Thematic Analysis

Thematic analysis of the interview transcripts yielded four primary themes: deliberately planning families, more is more, presenting polyamory, and living in a mononormative world. As per the nature of the research question, aspects of each of these themes focused heavily on relations with care providers and pregnancy care experiences, previously presented by Arseneau et al. (2019). However, significant sub-themes primarily pertaining to interpersonal relations, evolving identity, and stigma and barriers during the time of pregnancy and childbearing arose and have not previously been reported. These are the focus of this article.

### Deliberately Planning Families

Across interviews, research participants discussed the time and effort dedicated to making decisions regarding pregnancy, birthing, and parenting (Arseneau et al., 2019). Overall, participants indicated that many of the serious decisions they made, such as choice of care provider (Arseneau et al., 2019), were carefully weighed and impacted by their status as polyamorous. Study participants outlined having carefully considered pros and cons of numerous situations and manifestations of family structures in order to make choices that would be best for their futures and the futures of their children. These decisions often involved all partners and avoided making assumptions on others’ behalf. Decisions around roles and parenting and sexual health exemplify the conscious nature of decision making surrounding pregnancy and parenting in polyamorous relationships and households.

*Roles and Parenting* Perhaps the most salient aspect of the general theme “deliberately planning families” is that of discussing and deciding on parenting roles. Across all interviews, rather than assuming desire to parent, individual interest in parentage was considered and prioritized.

Our son is six weeks old right now. He was born in October, so I got pregnant in January. And how this might relate to polyamory is my other partner of two years, boyfriend, he is not interested in having children. He is not in any other relationship right now other than me and him. He enjoys children, likes being involved and in a child’s life but is not interested in making any of his own. (K1).

In many cases, this translated to relationship reconfiguration as becoming a parent was posited as a fundamental change in-personal identity. Not only were roles and titles salient when delineating responsibilities, they also sent a message to the world outside of the family unit. For example, in some multiple parent family units, each parent was referred to as mom/dad/mama/daddy/etc. and in others, non-biological parents who had perhaps joined the family after the decision to get pregnant were termed stepparents. Other families described non-parenting partners as taking on the role of auntie or uncle: individuals important to the child(ren)’s upbringing who did not make decisions on behalf of the child(ren).

As a result of the shift in roles and responsibilities, some relationships dissolved with the advent of the pregnancy, while others became more casual or even more committed. Having children (whether a first or subsequent child) was recognized by all as a time of great transition, particularly in relationship structure.

So as the pregnancy went on, I just started withdrawing from that relationship with (ex-partner). And then ultimately, I think it was in February that I told him that I thought it best if we split up. Just we wanted different things (A1).

How did you feel, (A4), about dating a new mom with a two-month-old? (Interviewer).

I don’t know. I had no idea what I was getting myself into. (A4).

And here you are two years later. (A1).

As described in the quotes above, relationship transitions occurring during pregnancy or in the early postpartum period were largely direct results of these important life events. As children tend to change nearly every aspect of one’s life, partners desiring different things were not uncommon. Some participants described a desire to focus on their “nest” and prioritize parent/child relations. Others explained that they were simply too overcome by their new babies to consider dating or fostering new relationships.

My nesting partner and my family are my priority and as much as I love my other partners, they take precedence, the long-term over the short-term. (J1).

*Sexual Health* As family and relationship structures varied, so did the elements of choice regarding biological parentage. In some cases, participants reported choosing which person with a uterus would carry a child and in others which individual’s sperm would be used to fertilize an egg. These decisions were further complicated by biology in cases where, for example, all individuals involved had uteruses.

I was going to be the one who was going to be pregnant because she didn’t want to be pregnant at all. I didn’t have strong feelings about being pregnant, but she had strong feelings about not being pregnant. (H1).

Just as participants’ behaviors were modified to get pregnant with certain partners, they were adjusted to not get pregnant with others.

This was also a little bit awkward for my relationship with my other boyfriend at the time because of course we had to be extra careful about not getting pregnant. I didn’t want to get pregnant with the boyfriend when I was trying with the husband. So that made a spontaneous sexual relationship more difficult. (I1).

Beyond promoting/avoiding conception, talk of protection from sexually transmitted infections and ensuing behaviors was common among participants. As all participants were sexually involved with one or more people, establishing STI screening schedules was quite relevant. Several participants argued that polyamorous individuals tend to be more open in having these discussions than non-polyamorous people.

As far as sexual health goes, polyamorous people tend to be a lot more comfortable talking about STIs and protection in general, which I think is a really positive thing about polyamory and something that I think the general public should just become more comfortable talking about. (A1).

Back when I started more actively being poly shall we say, I had to talk to my doctor about it because I wanted to get tested more often, and he was willing to help me get the testing I needed. (I1).

In one participant’s formal employment, they were frequently responsible for having conversations with people about their sexual health, particularly about sexually transmitted infections. This, she believed, provided her with a contrast to how open communication was in her own relationships.

So I talk to a lot of people about their sexual health and their partner situation, and I am grateful nearly every day for the honesty that we have because some people are in situations that are not good, where they have no clue what their partners are up to. (E1).

### More is More

There was a resounding consensus among study participants that having more partners led to feeling more supported, particularly surrounding pregnancy and childbirth (Arseneau et al., 2019). This support came in multiple forms including logistical assistance such as scheduling and financial support, playing to individual strengths and weakness in relationships, as well as the provision of mental and emotional encouragement and relief.

So basically to me, poly is it’s not making the best of what you have, it’s taking the best of what’s available around you. And it’s not I’m making due, it’s how can I make these people’s lives better? And what can we do to enhance our lives, to enhance the world around us? (B1).

Participants felt that they received support from their multiple partners and from their partners’ partners. Participants described how having multiple partners allowed for them to have “more to take” or draw from in their relationships, and in return allowed them the energy to have “more to give” to respective partners.

*More to Take* During the time of transition that is pregnancy and childbirth, having the attention, affection, and support of multiple partners had positive impacts on the lives of pregnant participants.

But it also compounds the amount of love and support you get too which is my favourite part. (C1).

With more than one or two adults present to contribute to raising children, individuals were able to focus on their strengths while other partners contributed where they may have had weaknesses. One participant in a blended family explains:

But the one thing that I think that really works with our family is we all have this weird dynamic. I don’t do well with kids, says the girl that has them all. (B1).

In this particular family unit, the birthing person (B1) focused on what she termed the “mom stuff,” while her partners did the “thinking stuff” and the “kid stuff.”

(B3) likes playing games. She has the patience. I have no patience. The kids wanted to play this stupid game and the three of them sat around and played it… (B2) is very logical and he is great with science and math and all things that require thinking, again, not my forte… So, I do the mom stuff. I do the grounding, the chore giving, the allowance giving, I make the appointments and I do the tuck-ins at night. (B1).

Maintaining positive lines of communication within polyamorous relationships also made it possible for folks to step away from interactions and embrace time alone when available. It also meant that individuals in relationships could count on others to “fill in” for them when needed. Logistically, during pregnancy, this often meant that if the partner who typically attended appointments and ultrasounds was unavailable, they could feel comfortable knowing someone else was present to support the pregnant person. Several participants noted that even partners who did not intend to parent were invaluable throughout the pregnancy and early postpartum period.

I think being polyamorous was really nice during the pregnancy because that first echo I wasn’t there, and I felt bad about that. I was sad, but it was the one trip I couldn’t really change and I was very glad that her girlfriend was there for her, that she had somebody there so she had the support that she needed that I couldn’t provide at that time… She offered a few times if I couldn’t make it, so that was really cool. So, I think that’s a big advantage about a poly relationship. (H2).

*More to Give* Though having multiple partners frequently provided support, it also meant that participants had more people to take into consideration when making decisions and a greater number of people to care for. These demands were not insignificant, especially in the context of pregnancy.

And so, this pregnancy has been, so far, physically and just emotionally tougher and just all the responsibilities of just transitioning to being an adult, like working and then having to cook dinner and dealing with finances and just life is—(D1).

Not to mention our other people that we’re trying to support. (D2).

Mostly (D2)’s other people. (D1).

That we see. It’s a lot. It’s a lot sometimes. (D2).

Study participants frequently mentioned the importance of including partners in their lives and experiences to the extent that they wanted to be included. Being able to play to individual strengths in adult relationships and in childrearing meant that there was less burden on the individual and greater collective responsibility. Each person has an important role to play. Polyamorous relationships provided reinforcements to everyday life.

We all have our little rules and even things like doctor’s appointments. He’s down here with me this week because (B3)’s at home with the kids. We all have our places and whatever, but it’s talked out and we just make sure that everybody kind of is involved and everybody has a role and you don’t feel left out. (B1).

The presence of multiple people meant that, when needed, partners could advocate for each other and their needs. Logistically it meant that pregnant individuals could rely on partners to fill roles they normally might have but could not at the time.

Yeah. I couldn’t vocalize what I wanted, so he ended up having to be advocate for what we wanted which he did. He stepped up. (A1).

So, when I actually went into labour, [B3] came over to my house where I still lived with my ex-husband and took care of the kids. (B1).

And we’re kind of lucky because being in a triad it’s simpler and we’re all in one household, one combined house. So, when a decision comes up for an appointment or something, can you go? No. Can you? Okay, that works. It’s very quick. (B2).

### Presenting Polyamory

The third theme which came about in a number of ways throughout the interviews is presenting polyamory. The participants of this study frequently described whether they did and how they shaped presenting as polyamorous in various settings with various people (Arseneau et al., 2019). Overall, it was clear that choosing to be “out” as polyamorous meant a complex and unceasing series of actions and decisions.

*Coming Out* How individuals presented themselves or did not present themselves as polyamorous was an individual decision informed by space and time. Depending on the various identities occupied by participants, the safety of individuals and their families varied in the context of presenting as polyamorous. Impacts on children’s relationships, job security, relationships with family and friends, among others were both conscious and subconscious aspects of decision making. Often, “coming out” as polyamorous also meant coming out as a different sexual orientation than their friends/family previously assumed them to be.

I don’t know. I’m asexual. Actually, they ask me if I’m having sex with more than one person. The answer is no so it doesn’t come up. They’re usually surprised that I’m just not sexually active because I’m “ace” and then I usually get more flack about that than having multiple—well I used to have multiple romantic partners. I’ve only got one right now. But I know my girlfriend does disclose everything and she’s had people try to diagnose her as bipolar or hypersexual. (J1).

I told my parents and they were kind of like, oh. Because one of my partners was a woman, so suddenly they found out I’m bi and that I have more than one partner but they didn’t really like my other partners anyways. (J1).

A constant risk/benefit analysis took place in participant decision making about disclosure. When asked about choosing whether or not to disclose to health care providers specifically, participants often remarked that they decided not to but would have had it become medically relevant. Decisions were shaped by social situations and power dynamics. Threats to personal safety, employment, child custody, and relationships were all mentioned on multiple occasions.

And it’s a little bit tricky because over the years our experiences have been really mixed and variable. Some really good things have come out of telling certain people. And I also feel like we’ve also lost some friendships and people have been really weird and judgmental and had all sorts of assumptions and so to me it’s not worth it. (E1).

And I would say that the only remaining challenge for me is still the being socially open about it. (E2).

The public judgement towards—(E1).

The judgement towards it, yeah. (E2).

Really, the type of relationship that we’re in and having, along with the fact that there are also children involved, potentially. (E1).

Often precludes people from being transparent about the nature of their relationships (E2).

How and whether individuals presented themselves as polyamorous was also shaped by the nature of relationships with those being disclosed to. Participants occasionally indicated relationships with families and friends becoming strained or ending. That said, not all disclosures had negative impacts or any significant impacts at all.

I think a lot of interfaces between in sort of a non-traditional relationship and specifically how you deal with your family around that because your family might be a racialized minority or they might be immigrants like my parents, or they might be just very religious, or conservative, or whatever it might be. Most people’s families are not going to be like, oh, you two have multiple other partners? That sounds so lovely. (E2).

Participants who present socially as polyamorous described also having to present or define polyamory to others. Their decisions were shaped by public response and the burden of answering questions about polyamory. Individuals often expressed fear of misrepresenting polyamory or being tokenized by those asking.

You might have to draw a diagram. One of the other questions I always get too is like well, how is that any different than cheating? (C2).

I find everybody’s nosy. I have a few Trans friends and when I’m out with them, everybody without a doubt is like so, tell me about your pants. Not your business, same thing as my bedroom, not your business. (D2).

And I find most people don’t mean anything by it. It’s that childish curiosity, right? (D1).

Because sometimes when people ask, they then look genuinely embarrassed when I give them a saucy answer. They’re like, oh, oh, I didn’t even realize what I just asked. (D2).

As disclosure was clearly much more complex than simply saying “I am polyamorous,” interviewed participants tended to describe taking one of two approaches. These approaches, which focused either on social monogamy or social justice, could have serious repercussions for individuals in their relationships not only with those to whom they were/were not disclosing, but also on their relationships with their partners. By not disclosing, partners often were not introduced to important people in participants’ lives.

*Social Monogamy* The first approach, social monogamy, was generally adopted by individuals who did not want to/felt they could not present socially as polyamorous and therefore “passed” as monogamous in social situations. These participants worried not only about the impact of being “out” on their own lives, but on polyamorous people as a group.

So [K2]’s out about it to his close friends and he has now told his parents about the polyamorous situation, just kind of out of necessity and him wanting to be honest with them. But to most people, it looks like he is monogamous. (K1).

And I’m kind of worried that I won’t give the best answer that I would like to. So if I were to bring it up and people were to ask about the family dynamic how it works or just say something that sounded judgy to me, I’m worried about getting upset and incoherent and not being able to explain myself. And I feel like I would be upset if I didn’t do it justice. I think it’s important to present polyamory the way it is, like I feel it’s important for people not to come away with misconceptions. And I think I would be upset if I were not to represent it as I think it should be, if that makes sense? Like if you’re in a discussion with someone online, you’re able to think about how you want to explain it and write it out so that you’re satisfied with the explanation and in person that can be more difficult. And I think, in particular with me with anxiety, it just always seemed easier not to talk about it for that reason. (K1).

*Social Justice* In contrast to those individuals who generally presented themselves as socially monogamous, a group of participants described discussing their polyamorous identity as a form of social justice. These folks were comfortable with having lengthy conversations about polyamory and wanted to help in normalizing it. Some participants who presented themselves in this way felt that presenting this facet of their lives openly allowed them to be open about other parts of their identities.

We’re here and we’re queer pretty much. We’re loud and proud. (D1).

I walk around the neighbourhood in a froofy dress. (D3).

We’ve pretty much embraced our weirdoisms. (D1).

Our basic principles and the differences is in this study we’re involved in, [E1]’s like this study’s so great. It’s anonymous. It’s anonymized. I get to participate in it and talk about poly and then I get to add to the literature and finding stuff about poly and pregnancy. It’s really great and awesome. And I was like yeah, it’s super awesome in that way because now I get to tell all the friends and people that I interact with that I’m part of this, whereas she wouldn’t talk about it. She would just know that she’s a part of it and that’s kind of like our different perspectives towards things. Now, everybody that I talk to, I can say that I’m contributing to this. But I will have that discussion, whereas she would not have it as openly as I might. (E2).

As exemplified in the above quote, even within family structures individuals described significant differences in how they approached presenting polyamory. Furthermore, how much detail individuals provided when describing their relationships depended on with whom they were communicating and the circumstances surrounding communication.

### Living in a Mononormative World

Through each stage of life surrounding pregnancy and childbearing presented by participants, polyamorous individuals faced difficulties navigating social systems which often privilege monogamy (Arseneau et al., 2019). The struggles with mononormativity (the assumption that people are monogamous), for participants, were often compounded with experiences of heteronormativity, racism, fatphobia, and ableism among other issues. Many of the families and individuals interviewed belonged to multiple minority groups and were able to account myriad experiences that were rendered more complex due to these intersecting identities.

*Intersecting Identities* From navigating medico-legal systems to everyday social interactions, it was clear that the folks interviewed had to constantly contend with assumptions of monogamy. In particular, participants spoke of how their polyamorous identity intersects with other aspects of their identity to complicate daily life. For example, one participant spoke of how their sexual orientation, polyamorous identity, and race intersect to present unique challenges.

The polyamorous relationship between homogenous racial groups is one thing and as soon as you start to mix it up, that becomes another and a new dynamic in itself, too. And it’s very hard to predict what exactly the nugget of challenge in that is. There’s a lot of moving parts in it. It’s got the whole list. It’s got the gender. It’s got the sexuality. It’s got the orientation. It’s got the gender expression. It has the race. It has the age. All those jumble it out, throw it down and you could have a real mix of challenges…and there’s no cookie-cutter kind of way. (E2).

I felt like I had to fight a lot about being fat and pregnant. I fought a lot with every provider I came into contact with. I fought with my anesthesiologist consult. I fought with the initial OB that I had a consult with. I didn’t have to fight with my midwives that much, but there were a couple of things that kind of weren’t cool. (F1).

Participants also spoke of the impact of their polyamorous identity on social relationships when a power dynamic is present. This was especially true related to employment.

I’m trying to be a bit more open with work and stuff like that, and so it is very tricky because I’m not a manager or a middle manager and I feel like these power differentials can affect my ability to earn a living. And so I have to be careful in that sense, but at the same time, I don’t want to be persecuted for having loving relationships that people don’t understand. And I’m more than willing to have the conversation if you want to have the conversation, but if you’re going to want to be prejudice and judgmental, then I can’t do anything about that. It’s a fine balance. (E2).

*Power Struggle* Our interviews included several lengthy discussions of the barriers faced by polyamorous pregnant folks and their families within the healthcare system during pregnancy and birth. These were largely discussed previously in Arseneau et al., 2019. Not only did issues with assumptions of monogamy arise in issues related specifically to health, but also in all issues surrounding interactions such as experiences of physical space in hospitals and clinics, discussions with administrative staff in these centers, and the need to frequently “come out” to new care providers.

…there are never enough spaces for parents’ names on stuff, so a lot of times I always have to put in for the dads I always put (F2), (F3), their last names and all their stuff. (F1).

Navigating a world in that is not set up to accommodate polyamorous relationships can have serious repercussions, particularly surrounding legal contexts.

So this is about something that the government’s not going to be able to do much about, but companies providing health care don’t provide health care for an arbitrary number of parents. It would be reasonable for me to pay more if I was going to try and cover three parents on my health care, but I can’t even do that. (F3).

Although for travel we always make sure that either one of us writes a letter in case they get asked at the border. We never have been. (F3).

You both write a letter if neither of you are present? (Interviewer).

Yeah, and the thing is too is that I’ve always been there. I don’t know what would happen if say one of them tried to take their not biological child, like what would happen? I don’t know what would happen. We’ve never been asked for such a thing, but then we present as a couple. (F1).

### What About the children?

While the impacts of each of the themes discussed above on children have not been addressed in detail, there are meaningful implications. Participants discussed how the difficulties that accompany navigating a mononormative world often lead to lack of partner or non-biological parent acknowledgment which in turn affects social interactions of parenthood.

Yeah, so we’ve started having some discussions about is this something that if E2’s boyfriend picks up [son] from daycare, what would we say and I say oh, we say he’s a friend, and E2 says that’s not quite fair (E1).

These boundaries and barriers had significant impacts not only on adult/child interactions, but also on how children navigated the world. Because of the stigma often associated with polyamory, children sometimes bore the brunt of judgment from adults.

That boy [who knew about us being polyamorous] wasn’t allowed to come to my son’s birthday because of who we are and what we do. It’s very wrong in [his parents’] eyes to the point where they wouldn’t even allow their son to come in our yard to have an outdoor party, which is very sad because they rode by during the party and stopped to say hi. (G1).

Despite these occasional negative experiences faced by children of polyamorous families, participants shared numerous ways in which they perceived their polyamorous relationships to provide benefits to their children. Logistic benefits such as having more caregivers and abstract benefits such as improved communication were mentioned frequently.

[My mother] was like, “I was a little worried about the kids, but the kids seem grounded and well-rounded and happy, and you’re happier than you’ve ever been…” (B1).

And I find that us being poly has made our teenagers a lot more willing to talk to us about—(D1).

Everything. (D2).

Everything. [Daughter] came out as I think I’m gay. Okay. And then this year we were at the parade and she picked up a pan pen. She’s like, so I’ve been learning stuff. Like, go you. And we talked about it a lot. (D1).

That was an icebreaker today at school. (D2).

It was an icebreaker today at school. Somebody noticed her pan pen and was like—(D1).

I came out yesterday! (D2).

So I find us being out and not really quiet about who we are is giving our children a lot of confidence to be their own little weirdo selves too, which is kind of nice. (D1).

## Discussion

Overall, participant experiences converged and diverged on a number of emotions, events, and thought processes throughout their pregnancy and birth experiences in the context of being polyamorous. Participants tended to describe many of their personal lived experiences as positive such as gaining support from partners and partners’ partners, feeling faithful to their own identities, and generally having positive childrearing experiences. In contrast, many of the stressors and negative impacts on their experiences came from external pressures and people outside of the family unit. These included stigma and prejudice surrounding disclosure, difficulties navigating medical and legal systems, and fears of how these external pressures might impact their children.

The data gathered through the interviews were extremely rich as people were very eager to share their experiences with the interviewers. Discussions branched significantly from the primary research questions in a multitude of ways allowing for some unexpected outcomes. While the initial goal of the research project was to focus primarily on the experience of pregnancy and birth, participants were keen to discuss their experiences of evolving relationships with partners and family members, experiences with other care providers over time, and hopes for the future among other subjects.

The compilation of the glossary of terms has provided a succinct yet meaningful way of distributing data from this study. Not only are participant experiences contextualized with the use of quotes to define each term, but the definitions and quotes were approved by participants prior to being made public. The glossary of terms has been made accessible to care providers, researchers, and the general public to better inform practice and interactions. As feeling pressure to perfectly convey polyamory when explaining the practice/identity was brought up by several research participants, having a resource to direct people to may help answer common questions and demystify relationships. Ultimately, the glossary allowed us to confirm that we were able to represent participant ideas in the ways in which they were meant to be explained.

As stated previously, there was a thread of deliberateness of actions throughout the interviews. Participants spoke of conscious decision making when choosing whether to engage in relationships, planning the conception of children and parenting roles, labor and birth experiences, and the practical aspects of parenting. Reczek (2020) terms this intentionality, in the context of gender and sexual minority people “planned pathways for family formation” (p. 310). Members of sexual and gender minorities often have more logistic barriers to having children from accessing assisted reproductive technologies to adoption and surrogacy, to the emotional tolls of these approaches. While few of the research participants of the present study faced such biological barriers to reproduction, they certainly did plan their family formation.

A recent study conducted by Pain (2020) examined the family practices of LGBTQ + polyamorists and considered how these families both strayed from and performed family practices in normative ways such as cohabitation, parenting, and marriage. Similar to the findings of the current study, participants had broad definitions of family extending from biolegal romantic/sexual relations and parenting relations. Pain argues that LGBTQ + polyamorist families “broaden conventional notions of family with their unique family structures and processes” (p. 284). Further, many of the participants of said study who indicated being in childrearing roles discussed the cooperative elements of raising children. Pain mentions the mentality of “it takes a village to raise a family,” which is echoed in the “more is more” theme of our research project. This is similar to Pallotta-Chiarolli et al.’s (2013) concept of collaborative parenting in which several adults contribute to raising children. Within the present study, not only were participants involved in multiple parenting families once children were born, but multiple partners were involved from conception or even earlier family planning discussions. Childbearing participants spoke of receiving support from their partners and their partners’ partners which in turn allowed them to reinvest that support in childrearing and in their own partnered relationships. In addition to have more time to reinvest in relationships with others, participants were able to ensure some free time to themselves.

The theme “presenting polyamory” spoke of the intersecting identities of the research participants and how these together framed how they presented or chose not to present themselves as polyamorous. Though the participants recruited in the current study were heterogeneous on the basis of sexual identity and relationship configuration, as in many previous studies the sample was predominantly white, cisgender, and highly educated. Researchers have previously hypothesized why individuals belonging to other minority groups are not represented in polyamorous literature. Two guiding hypotheses have been used to explain the lack of racial diversity, socio-economic status, and education level of participants in this nature of research: (1) lack of representation due to bias in sampling and accessing this population, or (2) folks from different backgrounds are not engaging in polyamorous relationships for a number of complex reasons (Sheff, 2013).

Recruitment for the present study was concentrated in social media groups and unfortunately would not have reached people who either chose not to be part of said groups or who did not have access to the groups. Though the inclusion criteria of our study were left intentionally broad to facilitate diversity of experiences, we failed to focus on recruiting disadvantaged groups. It will be important for future researchers to attempt to remedy this lack of inclusion, and we are committed to being more inclusive in sampling in future research endeavors. It is possible, however, that both hypotheses are true and that while people who belong to multiple minority groups are under-sampled, the groups are small. Researchers have previously hypothesized why individuals belonging to other minority groups are often not participating in research focusing on polyamory. These people may find themselves in already precarious situations where being “found out” as polyamorous could seriously jeopardize their lives and have powerful repercussions. Furthermore, being polyamorous may in itself be an act of privilege as people need to have time available to dedicate to multiple partners.

As discussed, previous research has demonstrated that lay perceptions of polyamory are often quite negative, particularly when children are involved (Séguin, 2019). These perceptions are likely fueling the fears some participants expressed in their hesitancy to disclose their polyamorous status to care providers. Individuals expecting an expectation to legitimize polyamorous relationships upon disclosure has been described previously in the literature (Dixon, 2016). The theme Presenting Polyamory demonstrates how this phenomenon is often approached by polyamorous people: either they are open and public about their status with a goal of leading the conversation or they avoid discussion that may be quite critical. Participants described fears of negative reactions from people at every level: from family and friends to medical practitioners. They described numerous challenges in interacting with institutions built on assumptions of heteronormative monogamous relationships. The common intersection between sexual orientation and relationship orientation described by participants added further complexity to said interactions.

Participants described planning pregnancies, parenting on purpose, and spending quality time with their children. In cases where participants had older children, they described these kids as well-adjusted, open minded, and effective communicators. Overall, the impact of being polyamorous, and potentially of having multiple partners, was a positive one on fetuses, babies, and children. Participants described being well supported by their partner(s) during the time of transition that is pregnancy.

The experiences of POLYBABES study participants interacting with care providers has been explored separately and in detail (Arseneau et al., 2019). The overarching message from this exploration is very comparable to the greater societal discussions alluded to in this paper: polyamorous folks are existing, living in family structures, and having and raising kids. There are immense benefits to being polyamorous while experiencing pregnancy and birth, but there is undeniable stigma and barriers across every aspect of health care—this is not exclusive to care surrounding pregnancy and birth.

Some limitations of this research project should be recognized. As the project was retrospective in nature and asked participants to consider experiences that occurred up to 5 years ago (or longer if speaking about experiences birthing and raising children who are now older), some details may have been forgotten or may have evolved over time. In the future, it would be informative to conduct a longitudinal study reporting experiences of pregnancy, birth, and the postpartum period in an ongoing way. Overall, the semi-structured nature of this project allowed for the compilation of a vast collection of data; however, more narrow questioning would provide a richness of data unattained here. This study should be considered as a foundation for further inquiry. Finally, though participants were located in several provinces, there was no representation from the Atlantic provinces, or from the territories. Future research studies should consider recruitment strategies that would be more effective in reaching these folks as statistically they certainly do exist.
